# National Insurance Act: The Prospect of the First Valuation

**Published:** 1913-12-06

**Authors:** 


### NATIONAL INSURANCE ACT DEVELOPMENTS.

### The Prospect of the First Valuation.

When the Chancellor of the Exchequer had designed the main outline of his National Insurance scheme, he very naturally sought the advice of some of the most eminent actuaries of our day in regard to the finance of the Bill. It was on the strength of the reports of these actuaries that the amounts of the benefits and contributions are as stated in the Act. One exception to this statement should, perhaps, be mentioned—namely, that the charge made in the weekly contributions to cover the cost of medical benefit was not subject to actuarial estimate, but was inserted at the direction of the Chancellor as equivalent to 6s. a year. Subject, then, to this one qualification, the relation of the benefits to the contributions in regard to their actual amounts was determined by the most expert actuaries, who were fully qualified in all respects to advise the Government on so important a matter.

A good deal of misconception exists as to the function of the actuaries in regard to the scheme. It has even been suggested in certain quarters that they were responsible for the structure of the Act itself, but the Presidents of both actuarial bodies—the Institute of Actuaries and the Faculty of Actuaries in Scotland—have taken occasion recently to point out that the Government are responsible for the scheme as a whole, and that the part of the actuaries concerned was merely to advise as to the financial soundness of the proposals. In tendering this advice, as may be seen from the reports which were published by the Government at the time, it was clearly pointed out that the estimates were based upon the results of past experience of sickness and invalidity that had been tabulated by the Manchester Unity of Oddfellows Friendly Society. It is true that a small margin of about 10 per cent. was retained for possible adverse variation in the actual experience from the standard rates of sickness that had been adopted. But in view of the fact that all profits, if any, are the property of the insured persons, no real hardship was entailed by this surcharge in the contributions. Rather, it may be said to have conferred a slight advantage, because, in the event of the actual sickness experience proving more favourable than the standard, the margin will possibly enable some substantial additional benefits to be granted, while, on the other hand, if the experience is unfavourable, the margin will to some extent mitigate the severity of any measures that have to be taken to preserve financial stability.

### Actuarial Estimates and Valuations.

The main point to which we now wish to direct attention is that the actuarial estimates were based on the assumption, broadly speaking, that the experience of the past, in regard to claims for sickness benefit on the friendly societies, would be reproduced in the future operations of the same and other societies as approved societies administering State-aided Insurance. There is no possibility of gainsaying this statement, as it is made abundantly clear in the actuarial reports that it is so.

Now every actuary insists on the absolute necessity for periodical valuations to be made—a process of stock-taking, as it were—in all matters that relate to actuarial estimates. In life assurance practice the valuations are usually associated with the pleasing duty of declaring a profit in the form of a bonus to policyholders and shareholders. In friendly society valuations, by way of contrast, the valuations have too frequently provided occasions to the valuers of pointing to deficiencies and suggesting measures for their eradication.

The necessity for periodical valuations will at once be appreciated when it is borne in mind that the experience of the past is taken as a guide to the future. However well an original estimate may have been made, it is well-nigh impossible even for the most far-sighted actuary to so read the future from his knowledge of the past as to err neither in excess nor in defect. The opportunity is therefore taken at the periodical valuations for revising past standards of measurement and for amending estimates already made.

Bearing these facts in mind it is not to be wondered at that the National Insurance Act makes provision for periodical valuations, and that elaborate precautions are taken in the Act for giving effect to the results that may be shown.

### The First Triennial Valuations.

As it is of paramount importance in a national scheme that financial stability should be absolute, care has been taken to require regular valuations, and it is expected that the first valuations will be made as at the close of the Insurance half-year ending in July 1915. The precise date is not stipulated in the Act, the requirement of Section 36 being that " A valuation of the assets and liabilities. . . of every approved society and of every branch of an approved society shall be made by a valuer, to be appointed by or with the approval of the Treasury, at the expiration of every three years dating from the commencement of the Act (July 1912), or at such other times as the Insurance Commissioners may appoint." It is clear from this that the framers of the Act had in view a system of triennial valuations, and, although the Insurance Commissioners are given the power to appoint either longer or shorter intervals, it is expected that on the first occasion, at any rate, the triennial period will be adopted. If this should prove to be so, it will at once be seen that very nearly half of that period has already expired, and little more than eighteen months will elapse before the valuations have to be made. It is, therefore, not too early to consider in the light of the experience of the past few months the likely results of the first investigation into the financial soundness of the approved societies.

### What is a Valuation?

It is necessary first of all to state, as clearly as possible, what is meant by a valuation of the assets and liabilities of an approved society. The assets consist not only of the sums standing to the credit of the society in its investment accounts on the date of the valuation, but also the future contributions to be received from its members in so far as these relate to the benefits the society is liable to pay to the members. The contributions paid by the members from week to week include allowances for medical and sanatorium benefits, but as these benefits are under the administration of the Insurance Committees and not under the control of the approved societies, the benefits will presumably be omitted from the liabilities on the one side of the account, and the equal and corresponding value of the future contributions applicable thereto will be omitted from the opposite side.

The liabilities of an approved society are its contracts to its members.

The value of the assets will therefore consist of two parts—namely, (1) the value of the sums invested, including the accumulation of the reserves credited to the society on account of members who entered above 16 years of age, and (2) the value of the future contributions.

So far as the invested funds are concerned the value placed thereon will probably be made to depend upon the quotation of the stocks on the Exchange on the valuation date, while the basis for valuing the future contributions, as also the liabilities, will be prescribed by the Insurance Commissioners.

It will at once be observed that in valuing both the future contributions and the liabilities actuarial considerations are involved, and it is for this reason that the valuers appointed will have to be persons with actuarial qualifications. There is every prospect, therefore, that the actual work of valuation will be in qualified hands and the results will bear the stamp of authority, but it is not yet clear that the same basis of valuation will apply to all societies alike or if varying standards will be permitted. It is thought that in view of the wording of Sub-section (2) of Section 36 that the same basis will apply to all societies, distinguishing necessarily between men's and women's societies, and to the various special classes of insured persons. In the event of the value of the assets being exactly equal to the value of the liabilities—a rather unlikely occurrence—the valuer will be relieved of the necessity of advising as to the disposal of surplus or as to the liquidation of a deficiency. It need hardly be said that if the value of the assets exceeds the value of the liabilities there is a surplus to the credit of the members, while conversely, if the liabilities exceed the assets in present value the difference is a deficiency which, although for the moment it may not be realised, will imperil the ultimate solvency of the society unless steps are immediately taken to set the matter in order. Space will not permit us this week to deal with the question of the distribution of surplus nor the liquidation of deficiencies when they arise, but we shall return to the matter in an early issue.

### The Opinion of an Expert.

As bearing on the results that are likely to be realised we would call attention to the weighty utterance of Sir Gerald Ryan—an ex-President of the Institute of Actuaries and a recognised authority in the City on financial matters—in his speech as chairman at the annual meeting of the Hundred of Samford Benefit Society held at Ipswich on November 21. This speech was briefly noted in the daily press and is printed verbatim in the *Insurance Record* dated November 28. It certainly deserves the closest attention of all who are interested in the well-being of insured persons.

After referring to the present depreciation in the price of securities and pointing out to the members the possibility of a realised loss on this account he turned to the question of the rate of sickness prevailing. "They now had," he said, "the experience of a considerable portion of 1913, and they found the sickness claims not only for 1912, but for the bulk of 1913, were considerably in excess of the standard to which the Society had been accustomed. They had, naturally, to inquire if there was any particular reason for that increase. Every step had been taken to prevent undue sickness claims, but they were nevertheless very much higher. He happened to know that 1912 was quite a healthy year, therefore the extra sickness in that year was not due to any temporary circumstances. It was, he thought, mainly due to the new conditions introduced into their friendly society system by the National Insurance Act, and he was afraid that those circumstances were of a permanent character." He then went on to refer to the part he had taken in publicly recommending friendly societies to seek approval for the purpose of administering State Insurance on the ground that they would benefit financially on their voluntary insurance from the reserves that were estimated to be set free. This expectation of the framers of the Act had not been realised because the members of the friendly societies, with very few exceptions, had chosen to pay the National Insurance contributions in addition to those paid voluntarily. By this means there has arisen the unfortunate condition of over-insurance. The members are actually better off financially in some cases when they are receiving sick pay than when they are employed on full time. No wonder then that malingering is rampant, and that the old friendly societies are the worst sufferers, for their voluntary funds, instead of being relieved, have extra claims to bear, and their State sections have all the weight of the higher sickness claims due to over-insurance.

---

NOTE.—Questions and Correspondence on all doubtful points are invited, and should be addressed to the Insurance Editor, The Hospital, 28 Southampton Street, Strand.

